# Structural evidence for intermediates during O_2_ formation in photosystem II

**DOI:** 10.1038/s41586-023-06038-z

**Published:** 2023-05-03

**Authors:** Asmit Bhowmick, Rana Hussein, Isabel Bogacz, Philipp S. Simon, Mohamed Ibrahim, Ruchira Chatterjee, Margaret D. Doyle, Mun Hon Cheah, Thomas Fransson, Petko Chernev, In-Sik Kim, Hiroki Makita, Medhanjali Dasgupta, Corey J. Kaminsky, Miao Zhang, Julia Gätcke, Stephanie Haupt, Isabela I. Nangca, Stephen M. Keable, A. Orkun Aydin, Kensuke Tono, Shigeki Owada, Leland B. Gee, Franklin D. Fuller, Alexander Batyuk, Roberto Alonso-Mori, James M. Holton, Daniel W. Paley, Nigel W. Moriarty, Fikret Mamedov, Paul D. Adams, Aaron S. Brewster, Holger Dobbek, Nicholas K. Sauter, Uwe Bergmann, Athina Zouni, Johannes Messinger, Jan Kern, Junko Yano, Vittal K. Yachandra

**Affiliations:** 1 Molecular Biophysics and Integrated Bioimaging Division, Lawrence Berkeley National Laboratory, Berkeley, CA USA; 2 Department of Biology, Humboldt Universität zu Berlin, Berlin, Germany; 3 Molecular Biomimetics, Department of Chemistry — Ångström, Uppsala University, Uppsala, Sweden; 4 Department of Theoretical Chemistry and Biology, KTH Royal Institute of Technology, Stockholm, Sweden; 5 Japan Synchrotron Radiation Research Institute, Hyogo, Japan; 6 RIKEN SPring-8 Center, Hyogo, Japan; 7 Linac Coherent Light Source, SLAC National Accelerator Laboratory, Menlo Park, CA USA; 8 Department of Biochemistry and Biophysics, University of California, San Francisco, CA USA; 9 SSRL, SLAC National Accelerator Laboratory, Menlo Park, CA USA; 10 Department of Bioengineering, University of California, Berkeley, CA USA; 11 Department of Physics, University of Wisconsin–Madison, Madison, WI USA; 12 Department of Chemistry, Umeå University, Umeå, Sweden; 13 Present Address: Institute of Molecular Medicine, University of Lübeck, Lübeck, Germany

**Keywords:** Bioenergetics, Nanocrystallography

## Abstract

In natural photosynthesis, the light-driven splitting of water into electrons, protons and molecular oxygen forms the first step of the solar-to-chemical energy conversion process. The reaction takes place in photosystem II, where the Mn_4_CaO_5_ cluster first stores four oxidizing equivalents, the S_0_ to S_4_ intermediate states in the Kok cycle, sequentially generated by photochemical charge separations in the reaction center and then catalyzes the O–O bond formation chemistry^1–3^. Here, we report room temperature snapshots by serial femtosecond X-ray crystallography to provide structural insights into the final reaction step of Kok’s photosynthetic water oxidation cycle, the S_3_→[S_4_]→S_0_ transition where O_2_ is formed and Kok’s water oxidation clock is reset. Our data reveal a complex sequence of events, which occur over micro- to milliseconds, comprising changes at the Mn_4_CaO_5_ cluster, its ligands and water pathways as well as controlled proton release through the hydrogen-bonding network of the Cl1 channel. Importantly, the extra O atom O_x_, which was introduced as a bridging ligand between Ca and Mn1 during the S_2_→S_3_ transition^4–6^, disappears or relocates in parallel with Y_z_ reduction starting at approximately 700 μs after the third flash. The onset of O_2_ evolution, as indicated by the shortening of the Mn1–Mn4 distance, occurs at around 1,200 μs, signifying the presence of a reduced intermediate, possibly a bound peroxide.

## Abstract (web summary)

Using serial femtosecond X-ray cystallography, we provide structural insights into the final reaction step of Kok’s photosynthetic water oxidation cycle, specifically the S_3_→[S_4_]→S_0_ transition where O_2_ is formed.

## Main

**Fig. 1:**
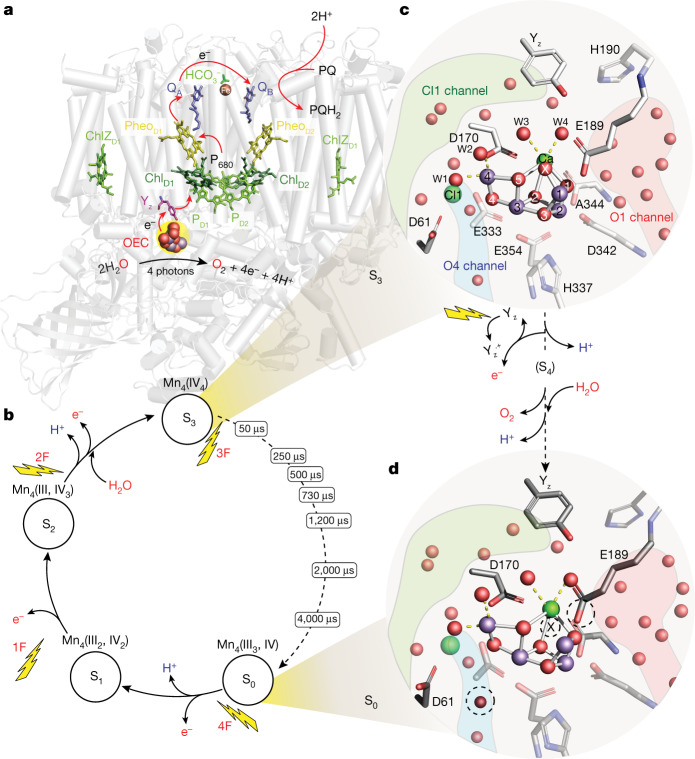
An overview of PS II and the electron donor site where water oxidation takes place. **a**, The structure of PS II with the membrane-embedded helices and the membrane extrinsic regions on the lumenal side of PS II shown in gray. The main electron transfer components are shown in colour, which include the reaction center chlorophylls (P680), pheophytins, acceptor quinones Q_A_ and Q_B_, redox-active tyrosine Y_z_ and the catalytic Mn_4_CaO_5_ cluster. The Y_z_ and Mn_4_CaO_5_ cluster are the cofactors of the electron donor site. **b**, Kok cycle of the water oxidation reaction taking place at the donor site that is sequentially driven by charge separations in the reaction center P680 induced by the absorption of photons (nanosecond light flashes, 1F–4F) in the antenna system of PS II. Room temperature X-ray crystallography data were collected at the time points indicated during the S_3_→S_0_ transition. **c**,**d**, The structure of the OEC in the S_3_ (**c**) and S_0_ (**d**) states and the sequence of events occurring between them. Mn, purple; Ca^2+^, green; O, red. W1, -2, -3 and -4 are water ligands of Mn4 and Ca. The relevant channels for water and proton transfer (O1, O4 and Cl1) are indicated as red, blue and green shaded areas, respectively. The dotted circles mark structural differences between the S_3_ and S_0_ states.

Serial femtosecond X-ray crystallography at X-ray free electron lasers (XFELs)^7^ enabled us to collect crystallography data of photosystem II (PS II) in real time as the reaction progresses at physiological temperature. The four photon-induced water-oxidation reaction in PS II (Fig. 1a,b) was initiated with multiple visible laser flashes. Using this capability, intermediate S-state structures (S_0_, S_1_, S_2_ and S_3_) have been studied^4,5,8–10^ that revealed the structural changes of the oxygen evolving complex (OEC) of PS II, which is a functional unit composed of the Mn_4_CaO_5_ cluster and its water–ligand environment (Fig. 1c,d)^2,11^. Recently, we collected snapshot data at several time points during the S_2_→S_3_ transition, the step in which one substrate water is introduced into the cluster. The study^6^ suggested the sequence of Mn oxidation, incorporation of an extra oxygen bridge (O_x_ or O6 in Suga et al.^10^) between the open coordination site at Mn1 and Ca (forming Mn_4_CaO_5_-O_x_ in S_3_), the potential entry path for substrate water and the proton release with its gating mechanism^6,12^. The XFEL studies also clearly established that the electronic and geometric structure of the OEC obtained by these measurements is unaffected by X-ray photoelectrons under the conditions used^6,8,13^.

In the current study, we investigate the oxygen evolving step of Kok’s water oxidation cycle (Fig. 1b), the S_3_→[S_4_]→S_0_ transition (Fig. 1c,d). In dark-adapted PS II samples, this transition is initiated by the third visible laser flash. The OEC is oxidized in this step from the all-Mn(IV) S_3_ state to the proposed highly reactive S_4_ state with formal oxidation states of Mn(IV)_4_O^⦁^ or Mn(IV)_3_(V). This initiates O–O bond formation and O_2_ release, and the now vacant binding site is filled by a new water substrate forming the lowest oxidation state of the cluster (S_0_). This multistep process, which also involves the release of two protons, has the longest time constant among the S-state transitions, and its kinetics depend on the species and sample preparation^14–19^.

To provide structural insight into this complex reaction step and specifically, the important interplay between the Mn_4_CaO_5_ cluster and its protein–water environment (Fig. 1c,d), we collected room temperature crystallography data of PS II at seven different time points during the S_3_→S_0_ transition, ranging from 50 μs to 4 ms after initiating this transition (Fig. 1b). All datasets have resolutions between 2.00 and 2.16 Å (Extended Data Tables 1 and 2).

The S_3_ state was populated by illumination of dark-adapted PS II microcrystals with two in situ visible nanosecond laser flashes (2F in Fig. 1b)^4^, in which the interval between flashes was 200 ms to account for acceptor quinone Q_A_ and Q_B_ kinetics and efficiently drive S-state transitions. The time points between the S_3_ and S_0_ states were generated by giving the third visible pump laser flash at various delay times (Δ*t*) before the crystals were exposed to the femtosecond XFEL pulse (the time labels in Fig. 1b). While our illumination protocol achieves the highest possible populations of particular S states, there is a higher mixing of S-state populations with increasing flash number due to intrinsic PS II-specific inefficiencies (‘misses’) (Methods)^20^. We model this distribution in a multicomponent model during structural refinement, with the ‘primary’ component being the centers that advance from the S_3_ to S_0_ state. The ‘secondary’ and ‘tertiary’ components are the known starting and end points: for example, the decreasing S_3_ population and at longer delay times, the increasing S_0_ population from centers that have completed the transition (Methods and Extended Data Table 3 have details). We note that the primary component at each time point may consist of a mixture of multiple structures, which are intermediates between S_3_ and S_0_. All results discussed below correspond to these refined primary components from monomer I (chains annotated as uppercase in the deposited structures).

## OEC and the Y_z_ region

**Fig. 2:**
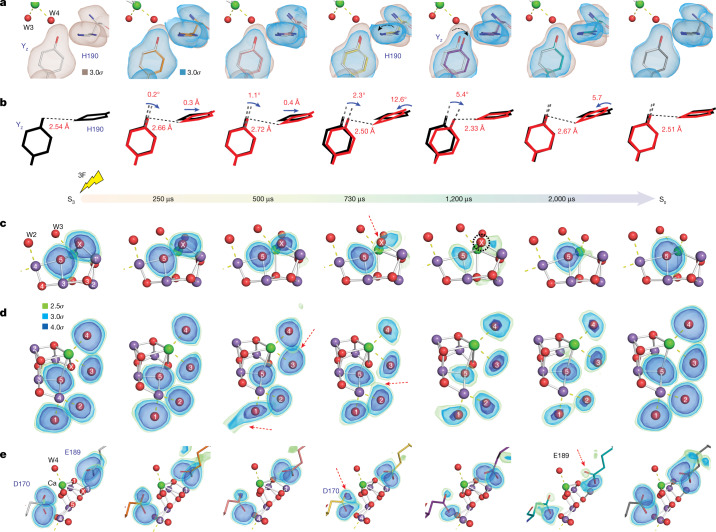
m*F*_obs _− D*F*_calc_ electron density omit map of key components of the redox active donor site of PS II at five time points along the S_3_→S_0_ transition as well as the S_3_ and S_0_ states. **a**, Residues D1-Y161 (Y_z_) and D1-H190. The omit map from the S_3_ state reference is shown in light brown for comparison with the time point data (blue). **b**, A simplified representation of the structural changes observed at the Y_z_ region. **c**, Omit density of atoms O5 and O_x_ of the OEC. **d**, Omit density of atoms O5 and the terminal water ligands W1, W2, W3 and W4 of the OEC. **e**, Omit density of carboxylate oxygen atoms of D1-E189 and D1-D170. All omit maps shown in **a** and **c**–**e** were generated by omitting the atom or residue of interest individually, and only the primary component (that is, the state that is advancing to S_0_) was used. Notable features are highlighted with red arrows and black dashed circle. All omit maps shown are contoured at 2.5*σ*, 3*σ* and 4*σ* using the colour scheme annotated in **d** for easier visualization. See also Supplementary Information Video 1.

**Fig. 3:**
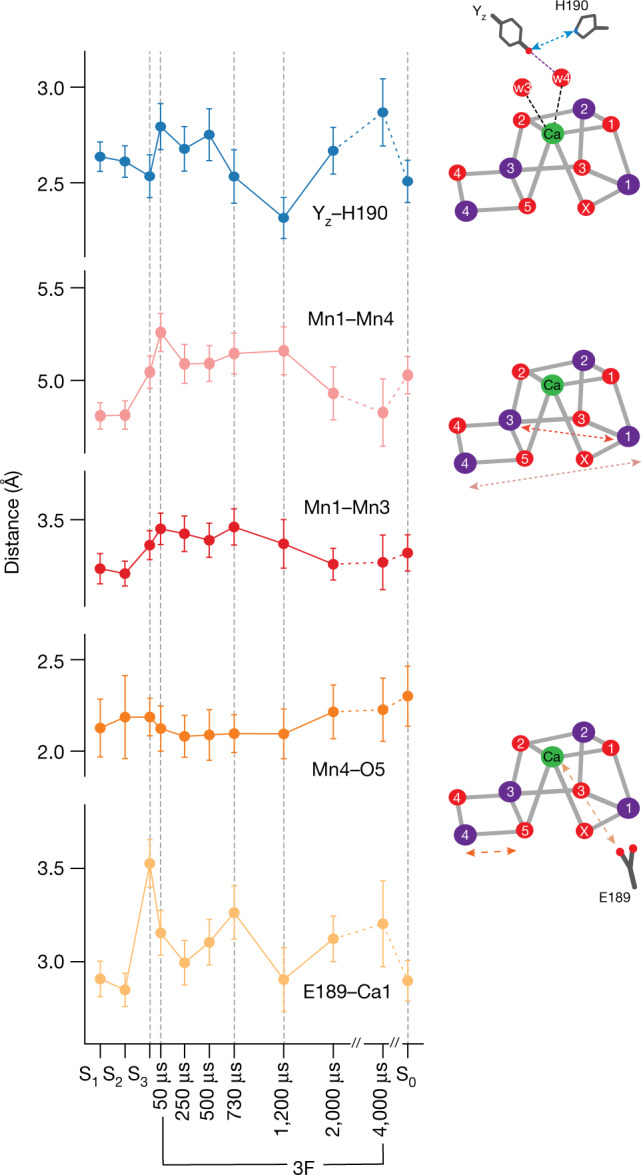
Distance changes between selected atoms/residues in the OEC during the S_3_→S_0_ transition. All distances are taken from the refined component of each time point (that is, the state that is advancing to the S_0_ state). Error bars are calculated from the end/rapid approach described in Methods and are an upper limit. Data here are shown as mean values ± standard deviation. The error bars for each time point were obtained from *n* = 100 independent END/RAPID refinements. More details about the END/RAPID procedure can be found in Methods. Dashed arrows in the schematics of the OEC on the right indicate the location of the individual distances. Mn is shown as purple spheres, and O is shown as red spheres.

Figure 2 shows the omit map density of selected atoms at the OEC and its surroundings of the refined population at the time points (Δ*t* = 250, 500, 730, 1,200 and 2,000 μs) after the third flash (we use the nomenclature of 3F(Δ*t* μs)). To visualize the sequence of events during the S_3_→[S_4_]→S_0_ transition, we follow the changes in three areas: Y_z_ and D1-H190 (Fig. 2a,b) and O_x_ and O5 (Fig. 2c), as well as the water ligands W1–W4 (Fig. 2d) and two carboxylate ligands of the Mn_4_CaO_5_/Mn_4_CaO_5_-O_x_ cluster that bridge between Mn and Ca (Fig. 2e). Additionally, selected atomic distance changes are shown in Fig. 3, including the earlier (50 μs) and later (4,000 μs) time point data.

The distance between Y_z_ and D1-H190 has been established previously to be an indicator of the oxidation state of Y_z_^6^. In the reduced state, a strong hydrogen bond between Y_z_ and D1-H190 leads to a short distance of about 2.6 Å. Upon oxidation of Y_z_ by P680^+^, the phenolic proton of Y_z_ is transferred to D1-H190, the distance increases to 2.8 Å and a movement of the His ring plane is observed (Fig. 2b). Our data show that the distance already increases between the S_3_ data and the first time point (50 μs), indicating that Y_z_ is fully oxidized by this time (Fig. 3). Thereafter, this distance remains constant until 500 μs and returns to the base level between 730 and 1,200 μs. Thus, the data demonstrate that ${{\rm{Y}}}_{{\rm{z}}}^{\mathrm{ox}}$ reduction by the Mn_4_CaO_5_-O_x_ cluster starts only after around 500 μs (Extended Data Table 4)^16,21,22^ and appears to be complete by the 1,200-μs time point. Additional distance changes between Y_z_ and His190 are observed at 2,000 and 4,000 μs, which may be due to the rearrangement of the hydrogen bonding network related to the last proton release but are not well understood currently. Interestingly, a shift in the position of a Tyr residue next to the special pair Chl upon light excitation was previously noted in time-resolved crystallography data of the purple bacterial reaction center and interpreted as originating from a change in the hydrogen-bonding interactions of this Tyr upon deprotonation^23^.

**Extended Data Fig. 1:**
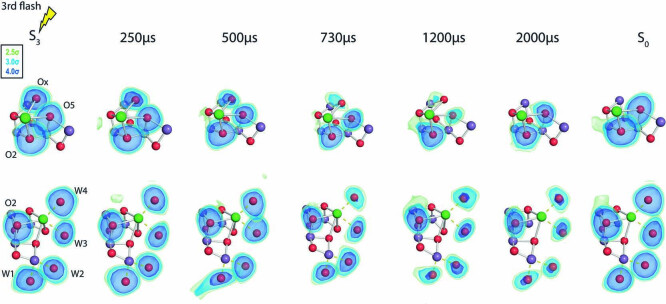
mF_obs_ − DF_calc_ electron density omit map of relevant atoms in the OEC for time points along the S_3_→S_0_ reaction. For comparison of the peak height level, the omit map of the oxygen atom, O2, is also shown. Omit maps for each atom were generated by individually omitting the atom only in the primary conformer in each dataset. The maps are shown at contour levels of 2.5, 3 and 4*σ*. (a) Omit maps of O_X_, O5 and O2 (b) Omit maps of W1, W2, W3, W4 and O2. A clear reduction in omit map peak height of O_X_, O5, W1, W2, W3, W4 is observed with respect to the reference O2 omit map between 730–2000 µs.

The extra oxygen O_x_, present in the S_3_ state, is lost upon S_0_ formation, indicating that O_x_ may participate in the O–O bond formation, and changes in its density probably inform on the onset of this process. For tracing the O_x_ population, O_x_ was eliminated from the OEC model, and its omit map density is shown in Fig. 2c. O5 was separately omitted, allowing comparison of their densities (see also Extended Data Fig. 1). The O_x_ omit density becomes asymmetric starting at 250 μs, with a clear reduction in intensity after 500 μs and dropping to the noise level between 1,200 and 2,000 μs, and O_x_ can only be modeled with a population of less than 20% in the 2,000 μs time point. The O_x_ intensity changes occur concomitantly with ${{\rm{Y}}}_{{\rm{z}}}^{\mathrm{ox}}$ reduction, indicating that the O–O bond formation occurs between 500 and 1,200 μs. During the entire S_3_→[S_4_]→S_0_ transition, the O5 density remains approximately constant, except for a decrease of its electron density at 1,200 μs.

Additional markers for the presence of O_x_ in the cluster are Mn–Mn distances. As reported previously, the Mn1–Mn4 distance increases during the S_2_→S_3_ transition due to the insertion of O_x_ (ref. ^6^). Figure 3 shows that the Mn1–Mn4 distance remains elongated in the 3F structures until 1,200 μs (Methods has a more detailed analysis) and then declines over the next 3 ms to attain the same value as seen in the S_1_ and S_2_ states. A similar trend is also seen in the Mn1–Mn3 distance. Thus, there is a delay between the onset of O–O bond formation (500–730 μs based on the changes observed for Y_z_ and O_x_) and the time when the Mn–Mn distances start to decrease (1,200 μs). The finding indicates that during this period, a water oxidation intermediate likely exists before the release of O_2_.

**Fig. 4:**
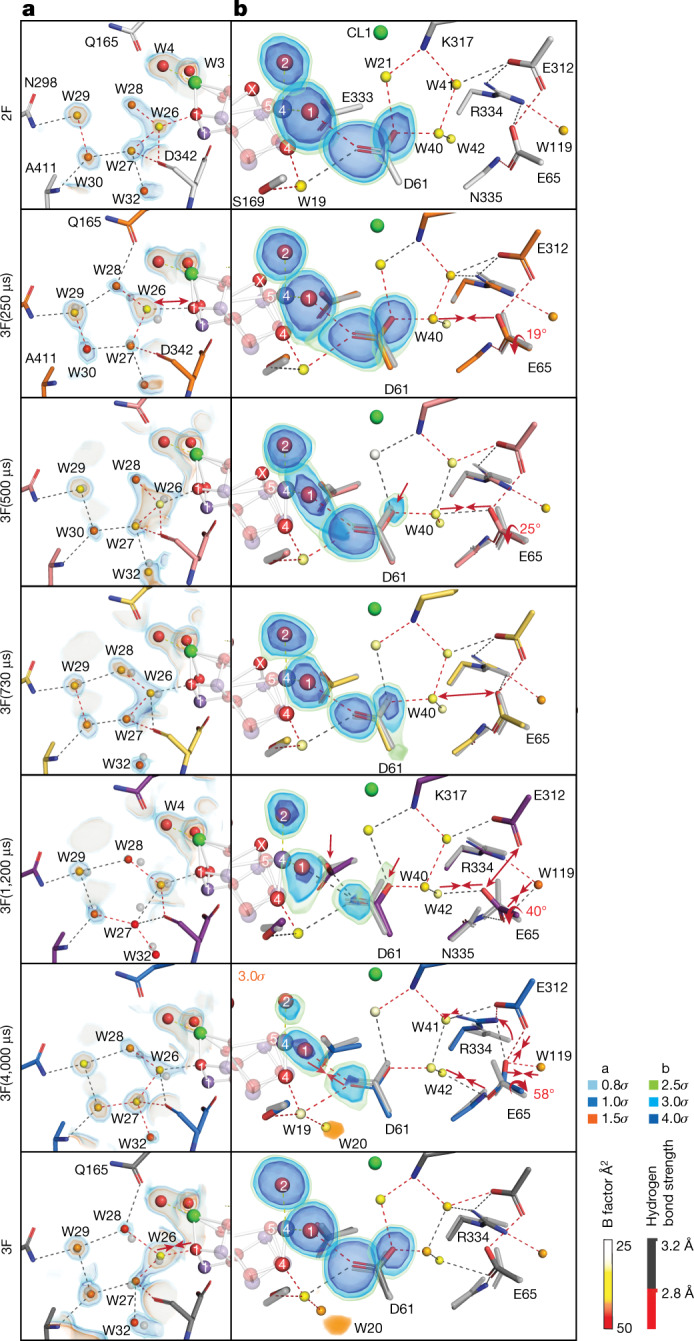
Structural changes in select regions of the water and proton channels of PS II during the S_3_→S_0_ transition. **a**, The terminus of the O1 channel near the OEC that includes the group of five waters (W26–W30) in this region. Overlaid is the 2m*F*_obs _− D*F*_calc_ electron density map contoured at 0.8*σ*, 1.0*σ* and 1.5*σ*. **b**, The O4 and Cl1 channels (branch A) that include the D1-D61 and D1-E65/D2-E312 region that is suggested to function as a proton gate. Overlaid are m*F*_obs _− D*F*_calc_ omit maps for W1, W2 and D61 shown at 2.5*σ*, 3.0*σ* and 4.0*σ*. Also shown is the *F*_obs_(time point) − *F*_obs_(2F) difference density map within a 1.5-Å radius of W19/W20/W48 in the O4 channel at 3*σ* (orange map). The observed rotation angle at the side chain of E65 at particular time points is calculated with respect to the corresponding side chain position at the 2F state. Major changes are highlighted with an arrow or dashed circle. All waters are coloured by their B factors according to the diverging colour scheme shown in the figure. Important hydrogen-bond interactions are shown with a binary colour scheme to indicate strength (distance < 2.8 Å is red and 2.8–3.2 Å is gray).

**Extended Data Fig. 2:**
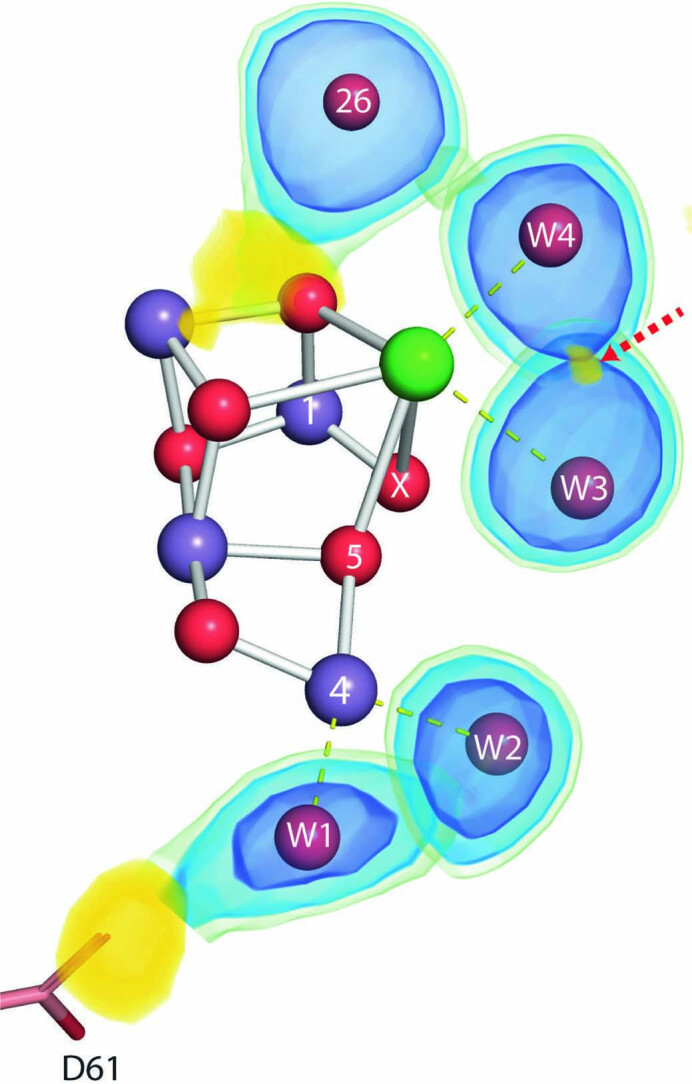
mF_obs_ − DF_calc_ electron density map near the OEC region in the 3F(500 µs) time point. The map is shown at a contour level of 2.5*σ* (in yellow). Electron density is observed at this level in-between W3 and W4 (see red arrow), possibly indicating a transient water motion that was also corroborated by the overlap of the omit map densities. For comparison, the individual omit maps of W3 and W4 are also overlaid at 2.5, 3 and 4σ (color scheme same as in Fig. 2 in main text). Stronger electron density is also observed around W1/D61 possibly related to motion involving a proton transfer as discussed in the main text related to Fig. 2. Electron density around the O1 is possibly related to changes observed in the W26-W30 (referred to as the ‘water wheel’ region) in this time point (discussed in main text related to Fig. 4).

Changes in shape and intensities are observed for the omit map density of the water ligands W1–W4, which are displayed in Fig. 2d. Specifically, a slight elongation of the O5 density toward W2 and a slight elongation of the W4 density toward W3 are observed at 250 μs. This latter trend continues, and at 500 μs, an overlap of the W3 and W4 densities is seen, with an m*F*_obs _− D*F*_calc_ peak (*F*_obs_ and *F*_calc_ are the experimental and model structure factors respectively, while m and D are weighting factors) at 2.5*σ* between W3 and W4 (Extended Data Fig. 2). At the same time point, the W1 density becomes extended toward D1-D61 and W19, indicating a higher mobility of W1. We speculate that all these motions are related to the deprotonation of the OEC and proton transfer toward the Cl1 channel (see the next section and Fig. 4).

At 730 μs, the O5, W2 and W3 densities become anisotropic, all pointing toward a region between these three oxygen atoms (Fig. 2d), which indicates increased mobility of these ligands. At the same time, the O_x_ density is also highly anisotropic. This movement of all four oxygen atoms is likely related to the formation of the water oxidation intermediate. We note that accurate modeling of the O_x_ position will require higher-resolution data. At 1,200 μs, the densities for all the terminal water ligands (W1–W4) and the bridging O5 become weakest. A comparison of these omit map densities within the OEC with that of the O2 atom, which is believed to not play a prominent role in the S_3_→S_0_ step, shows that the reduction of the density is specific to these five atoms (W1–W4 and O5) and O_x_ (Extended Data Fig. 1). The overlap of the O5, W2 and W3 densities is no longer observed at 1,200 μs.

At 2,000 μs after the third flash, the O5 omit map density is restored considerably compared with the S_3_ and S_0_ states, but omit map densities of the waters W1–W4 have not yet reached a similar level and remain elongated. We note a similar overlap of W1/W2 as observed at 500 μs. This could indicate the onset of the second proton release, known to occur in the S_3_→S_0_ transition, after the binding of a water that refills the vacant site formed by O_2_ release^21,24,25^. Interestingly, the elongated shape of the W3 density persists even in the S_0_ state, which we modeled previously with two possible positions of W3 (ref. ^4^).

Figure 2e shows that the D1-D170 and D1-E189 ligands, which both bridge between an Mn and Ca, change their conformation during O_2_ formation and release. Consistent with the high mobility observed for the W1 and W2 water ligands, the connection between D1-D170 and Ca appears to be weakened between 730 and 2,000 μs and is only fully restored at the S_0_ state (3F(200 ms)).

From 1,200 to 4,000 μs, several structural changes occur, which are reversed upon formation of the stable S_0_ state (3F(200 ms)). These include the increase of the Y_z_–D1-H190, Ca–D1-E189 and Mn4–O5 distances, as well as a decrease of the Mn1–Mn4 and Mn1–Mn3 distances. Most of these changes are indicative of O_2_ release and/or water insertion via the Ca ion^26,27^, possibly from the O1 channel (see below). This indicates that O_2_ release and refilling of the cluster by bulk water and resetting of the catalytic center occur over an extended timescale.

## Water and proton channels

PS II has several hydrophilic channels that extend from the OEC to the lumenal side of the thylakoid membrane^11,28–31^, and some of these are proposed to play a critical role in transporting protons and substrate waters during the catalytic cycle (O1, O4 and Cl1 channels are shown in Fig. 1)^24,32,33^. In our recent study of the S_2_→S_3_ transition^12^, we assigned the O1 channel, which extends from the O1 and Ca of the OEC to the bulk, to be a substrate water channel and the Cl1 channel, which extends from W1 and W2 of the OEC to the bulk, to be a proton release channel during the S_2_→S_3_ transition^6^. The S_3_→S_0_ transition also involves the insertion of one substrate water into the OEC and the release of two protons to the bulk; it has been suggested that one proton is released before the O–O bond formation and the other after the rebinding of a water molecule to the OEC^24,25,34^.

**Extended Data Fig. 3:**
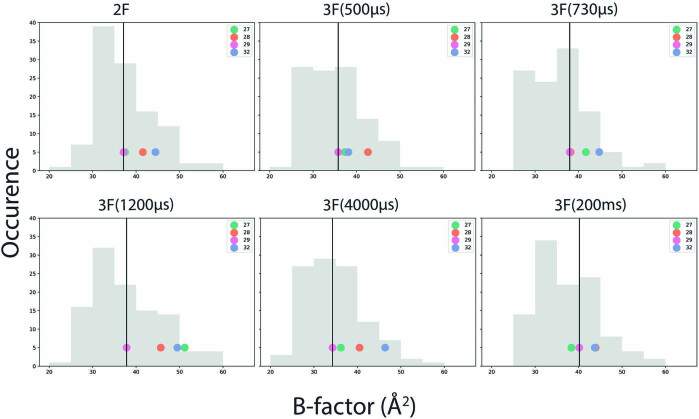
Comparing B-factors of key waters in the water wheel region of the O1 channel in the S_3_→S_0_ transition (W27, W28, W32) against a reference water (W29) for time points shown in Fig. 4 in the main text. The B-factor distribution of all the channel waters are overlaid for each time point for comparison. The y-axis values of the marker points for the 4 waters are just placeholders. There is a stark shift in the relative B-factor values of W27, W28 and W32 in the 3F(1200 µs) time point which coincides with the disappearance of O_X_.

Figure 4 shows the time point data for the O1 and the Cl1/O4 channels near the OEC during the S_3_→S_0_ transition. The electron density of water molecules in the O1 channel in the vicinity of the OEC changes substantially as shown in the 2m*F*_obs _− D*F*_calc_ maps (Fig. 4a), similar to what was observed during the S_2_→S_3_ transition^6^. Waters W27, W28 and W32 (Supplementary Table 1 has water numbering) have low electron density and high B factors (about 50 Å^2^) (Extended Data Fig. 3) relative to the more stable waters, such as W29 (B factor of about 37 Å^2^), in particular at 1,200 μs. We interpret this as an indication of the high mobility of these waters and hypothesize that this region could serve as the inlet for the substrate water that refills the OEC after the release of molecular oxygen^12^. We, therefore, propose that PS II uses the O1 channel for the substrate intake in both the S_2_→S_3_ and S_3_→S_0_ transitions. Among the group of five waters (W26–30) (‘water wheel’ in ref. ^6^), W26 shows high electron density throughout the transition, with a substantially elevated density at 500 μs. As W26 is within hydrogen-bonding distance to O1 of the OEC, this interaction may be important for balancing the charge on the cluster, when the OEC advances through the last oxidation step (that is, S_4_ state formation) and the subsequent four-electron reduction to form the S_0_ state.

Changes are also observed in the Cl1 channel. At 250 μs, the D1-E65 residue rotates by 19° toward W40, resulting in shortening the distance between D1-E65 and W40 by 0.3 Å (Fig. 4b). This is the time point when the O_x_ density starts to become asymmetric, which is even more pronounced at 500 μs (Fig. 2c). At 500 μs, the W1 omit map density also becomes elongated toward the region of D1-D61 and W19 (Fig. 4b). This coincides with a decrease of the D1-D61 carboxylate oxygen density that is within hydrogen bond distance of W40 and a shortening of the distance between W40 and D1-E65 by 0.5 Å due to rotation (25°) of the side chain. Consequently, a continuous hydrogen bond network is formed that connects the OEC to the D1-E65/D2-E312 region. We speculate that the changes are related to the first proton transfer from the OEC toward the Cl1 channel. These changes are reversed by the 730-μs time point (Fig. 4b). An early deprotonation event has also been suggested by other studies using different methods, with time constants that range from 50 to 300 μs (that is, before the last oxidation event; the transient S_4_ state formation)^15–17,21,24,34,35^.

At 1,200 μs, the D1-E65 residue rotates toward W40 for the second time during this transition, accompanied by a shortening of the W42–D1-E65 distance by 0.4 Å. Thus, a hydrogen bond network from the OEC to the D1-E65/D2-E312 region, similar to what we observed at 500 μs, is reformed at this time point. The D1-E65/D2-E312 distance elongates from roughly 2.6 to roughly 3.2 Å, which points to a substantial weakening of the interaction, likely forming a configuration that can accept the next proton. This change coincides with the time at which the O_x_ electron density decreases below the detection level in the omit map (Fig. 2), and the W28, W27 and W32 densities decrease in the O1 channel (Fig. 4a). We interpret this series of changes to be related to the onset of the recovery process of the Mn_4_CaO_5_ cluster (that is, initiated by the insertion of water into the OEC along with a deprotonation). At 2,000 and 4,000 μs, the side chain of D1-E65 is rotated almost 40° from its position in the S_3_ structure toward W119 (Fig. 4b). The distance D1-E65–W119 is around 2.5 Å, suggesting a shared proton or very tight interaction between these two groups. D1-R334 also moves by 20°, forming a hydrogen-bonding interaction with W41.

The changes around the D1-E65/D2-E312 region are indicative of the proton release to the bulk since they are reminiscent of what we observed for proton release during the S_2_→S_3_ transition^12^. In the S_3_→S_0_ transition, we hypothesize that the earlier changes of this region (250–730 μs) are related to the first proton transfer and that the later changes (1,200–4,000 μs) are related to the second proton transfer from the OEC to the bulk, through D1-D61^36,37^ via the rotation of D1-E65. Thus, the current result suggests that the D1-E65/D2-E312 region functions as a gate for proton release twice during the S_3_→S_0_ transition.

W20, which forms a tight hydrogen bonding network with O4 via W19 in the O4 channel, disappears during the S_1_→S_2_ transition and reappears in the S_0_ state^4^. Its return during the S_3_→S_0_ transition is therefore an indicator for the full recovery of the S_0_ state. The first clear indication of the return of W20 is found in the *F*_obs_(4,000 μs) − *F*_obs_(2F) difference map (orange density in Fig. 4b), and it was modeled at 40% occupancy in the 4,000-μs refined component. This implies that the W20 restoration happens in the later stage of the OEC recovery.

## Sequence of events during S_3_→S_0_

**Fig. 5:**
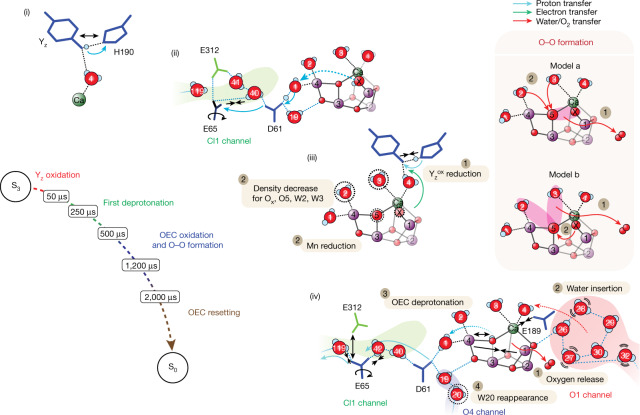
Schematic of the S_3_→S_0_ transition and proposed mechanism for O–O formation. The sequence of events (i–iv) leading to the first deprotonation event, the molecular oxygen release, the water insertion and the second deprotonation event. The OEC atoms are shown in purple (Mn), green (Ca) and red (O). The O1 channel is shown in red, the O4 channel is in blue and the Cl1 channel in green. The ligands of the OEC and the residues forming the water–proton channels are coloured based on the subunit they belong to (D1, blue; D2, green). Possible pathways for proton (cyan arrow), water (red dashed arrow), oxygen (red solid arrow) and electron (green arrow) transfer are depicted. Notable features are highlighted with black arrows. The right tan box shows the suggested models (model a and model b) for O–O bond formation. Oxygen highlighted with magenta indicates the candidate atoms for O–O formation.

The snapshots of the structures of PS II during the S_3_→S_0_ transition show the sequence and the progression of each of the events at multiple locations with different time constants. The structural changes can be broadly grouped into four sections with different onset times and kinetics, as shown in Fig. 5; they are the redox state changes of Y_z_, first deprotonation, OEC oxidation and O_2_ formation and the complete recovery and resetting of the Kok clock in the S_0_ state.

In the S_3_ state, all four Mn are formally in the (+IV) oxidation state. We note that oxidation of ligand instead of Mn (formation of oxyl or an oxo–oxyl bond) during the S_2_→S_3_ transition has been suggested in the literature^5^, but this is not in line with the room temperature X-ray emission spectroscopy (XES) data, which show the oxidation of Mn^6,13^. Upon the third flash, the oxidation of Y_z_ occurs by donation of an electron to P680^+^ after the charge separation at the reaction center chlorophylls. A distance increase between Y_z_ and D1-H190 is observed in comparison with the S_3_ state in 3F(50 μs) (Figs. 3 and 5(i)). This change is assigned to the ${{\rm{Y}}}_{{\rm{z}}}^{\mathrm{ox}}$ formation, which is known to occur within 30 μs after photoexcitation^38^, and related proton translocation between ${{\rm{Y}}}_{{\rm{z}}}^{\mathrm{ox}}$ and D1-H190.

The ${{\rm{Y}}}_{{\rm{z}}}^{\mathrm{ox}}$ formation triggers the first deprotonation event likely during the time period of 200–500 μs^15–17,24,34,39^. We observed the start of the rotation of D1-E65, which is proposed to be part of the proton gate, and the formation of the hydrogen bond pathway from the OEC to this region at 250 μs. Along with these changes, the electron densities at W1 and D61 become more prominent at 500 μs (Figs. 2d and 4b), potentially related to a proton release from the OEC to the proton gate residues D1-E65 and D2-E312 (Fig. 5(ii)).

In the early stage of the 500- to 1,200-μs period, the last oxidation event (transient S_4_ state formation, with Mn(IV)_4_O^⦁^ or Mn(IV)_3_(V)) occurs, and subsequently, the reduction of Mn takes place (Fig. 5(iii)). The O–O bond formation should be triggered by this final oxidation event of the OEC to the potentially short-lived S_4_ state. The change in distance we observe for Y_z_–D1-H190 between 500 and 730 μs suggests that the reduction of ${{\rm{Y}}}_{{\rm{z}}}^{\mathrm{ox}}$ takes place during this time, through the electron transfer from the OEC to Y_z_. After the transient formation of S_4_, the four-electron reduction may proceed in one step with the O–O bond formation and immediate release of O_2_ or in two steps with the presence of an intermediate before the release of molecular oxygen from the OEC. In the latter case, a peroxo species formed by an initial two-electron reduction appears most likely as an intermediate.

Our data show that there is a delay between the onset of O–O bond formation (500–730 μs) as indicated by the Y_z_–D1-H190 distance/rotation and the decrease of the O_x_ electron density and the onset of the O_2_ release supported by the Mn1–Mn4 distance contraction (1,200 μs). This onset time for O_2_ release is also in line with studies of O_2_ evolution^22,40^. The delay indicates that there is an intermediate state, possibly a peroxide-like species, pointing toward the two-step electron reduction mechanism.

Several O–O bond formation sites have been proposed in the literature based on theoretical studies (Fig. 5)^3,41–49^. Among these, O5–O_x_ best account for our data because of their proximity and the reduced occupancy of O5 around 1,200 μs (Fig. 5, model a). However, two other possibilities in which O5 reacts with either W2 or W3 and O_x_ replaces O5 cannot be excluded at this time (Fig. 5, model b). While other mechanisms that do not involve O5 cannot be ruled out, there is no clear evidence to support those in the current data.

At 1,200 μs, the O_x_ omit map density is below the 2.5*σ* threshold, indicating that a predominant fraction of O_x_ has shifted from its original position in the cluster. The contraction of the Mn1–Mn4 and Mn1–Mn3 distances starting at this time point suggests that the onset of O_2_ release happens around this time. Once O_2_ is released, refilling of the cluster with a new substrate water seems to occur immediately. This is based on the observation that there is no missing oxygen density besides O_x_, although the omit map densities of all the terminal waters (W1–W4) and bridging O5 are weakened at 1,200 μs. The data support that the O_2_ release and refilling of the site are highly coordinated and occur likely via a terminal water already ligated to the OEC.

At both 2,000 and 4,000 μs (Fig. 5(iv)), O_x_ density is within the noise level, which implies that Mn1 becomes predominantly five coordinate. Mn4 is six coordinate, although the Mn4–O5 interaction is weak (2.2–2.3 Å), suggesting that O5 may be a hydroxide. The Ca–D1-E189 distance is still more elongated at these time points than in the S_0_ state. Other slow recoveries are observed in the Y_z_ region (Fig. 2b), the ‘water wheel’ (Fig. 4a) and the proton gate regions (D1-E65/D2-E312) (Fig. 4b). We hypothesize that a water from the ‘water wheel’ region in the O1 channel, similar to the S_2_→S_3_ transition^6,12^, replaces the terminal water ligand of the OEC. The changes in the proton gate region might indicate the deprotonation of the newly inserted water^36^. Concomitantly, the amino acid coordination environment, the hydrogen bonding network around the OEC and the waters in the channels reset to the S_0_ state. This includes recovery of W20 in the O4 channel, which is proposed to be involved in proton release during the S_0_→S_1_ transition^4,50^.

In the current study, room temperature snapshots of PS II structures through the final step of Kok’s clock (S_3_→[S_4_]→S_0_) reveal details of the molecular processes for photosynthetic water oxidation. Until now, these processes were interpreted largely based on kinetic studies. Importantly, the results reported here provide experimental support for a two-step reduction mechanism of the Mn_4_CaO_5_-O_x_ cluster upon the O–O bond formation and O_2_ release with a transient intermediate, most likely a bound peroxide. This is a major step forward toward understanding the chemistry of the water oxidation reaction. The results also show how biological catalysts, such as the OEC in PS II, enable multielectron/multiproton reactions through the interplay between the metal center, the protein environment and the water network. The active role of the microenvironment in natural enzymes provides inspiration for how to control such reactions in artificial photosynthetic systems that can be made from earth-abundant elements.

## Methods

### Sample preparation

The X-ray diffraction measurements of 20- to 60-µm crystals prepared from PS II dimers of *Thermosynechococcus vestitus* (previously named *Thermosyncechococcus elongatus*) were performed in 100 mM 2-(*N*-morpholino)ethanesulfonic acid, pH 6.5, 100 mM ammonium chloride and 35% (wt/vol) PEG 5000 (refs. ^51,52^). PS II crystal suspension, at about 0.5–1.2 mM chlorophyll (Chl) concentration, was loaded into a syringe (Hamilton gastight syringe, 1 ml) and dark adapted for 1 h before data collection. Membrane inlet mass spectroscopy (MIMS) was used to determine the O_2_ evolution, turnover parameters and S-state populations^4,6^. The PS II crystals showed no Mn (II) contamination based on XES and electron paramagnetic resonance measurements^53^ and exhibited an activity of 2500 ± 100 μmol O_2_ (mg(Chl) × h)^−1^.

### Sample injection and illumination

Acoustic droplet ejection^54^ was used in combination with the Drop-on-Tape sample delivery method^55^. For capturing the stable intermediates S_2_, S_3_ and S_0_, each droplet of the crystal suspension was illuminated by 120-ns laser pulses at 527 nm using an Nd:YLF (yttrium lithium fluoride) laser (Evolution, Coherent) at Linac Coherent Light Source (LCLS) or by 8-ns laser pulses at 532 nm using a combination of two Nd:YAG (yttrium aluminium garnet) lasers (Minilite, Continuum) at Spring-8 Angstrom Compact free electron Laser (SACLA) via three fiber-coupled outputs with a delay time of 200 ms between each illumination and of 200 ms between the last illumination and the X-ray probe, similar to what was used previously to accommodate the acceptor quinone Q_A_ and Q_B_ kinetics and efficiently drive S-state transitions^4,6,55^. We implemented a feedback control system of the belt speed and deposition delay, and the flashing delay and droplet phase were adjusted accordingly^55^. To achieve time delays shorter than 200 ms between illumination and the X-ray probe, a fourth ‘free space’ laser was utilized. This was either an Opolette 355 LD laser (Opotek, 530-nm wavelength, 7-ns pulse width) at the macromolecular femtosecond crystallography/LCLS instrument or an NT230 OPO laser system (530-nm wavelength, 5-ns pulse width, EKSPLA Co.) at SACLA. This free space laser was triggered to be synchronized with the X-ray pulse with an adjustable delay that was set between 50 and 4,000 μs for this study. The laser was guided with optics to the X-ray interaction spot, and its position was fine-tuned for each delay time to ensure that the laser spot position coincides with the position of the sample droplet at the selected delay timing. At the XFELs, a light intensity of 120 ± 10 mJ per cm^2^ was applied as O_2_ evolution was found to be saturated at 70 mJ per cm^2^ for the dimensions and concentrations of samples used in our experiments^4^. A light intensity of 120 mJ per cm^2^ corresponds to about 140 photons absorbed per PS II monomer in the front 5-µm layer of the crystal and approximately 9 photons per PS II monomer when assuming a 60-µm thickness of the crystal (which is the upper size limit of the crystals used in this study) for the back 5-µm layer. This photon density ensures saturation over the entire crystal volume, even in the case of two crystals stacked on top of each other in the laser beam. Given a minimum pulse length of 5 ns and 35 Chl per PS II monomer, the light intensity used averages to 0.8 photons per (Chl and nanosecond) for the front and 0.05 photons per (Chl and nanosecond) for the back part of the crystal. If a PS II center is undergoing charge separation, additional photons absorbed by the internal antenna Chl are rapidly dissipated in the form of fluorescence with an average fluorescence lifetime of around 0.5–1 ns, hence preventing any overexcitation of the reaction center or causing any heating artefacts.

### X-ray data collection

The crystallography data were collected at various facilities, and details are listed in Supplementary Table 2. The experimental beam conditions and detector configurations used to collect each dataset are also tabulated. The sample was delivered into the X-ray interaction region using the previously described Drop-on-Tape setup^55^. Illumination conditions for populating different S states are detailed in ref. ^4^.

### X-ray diffraction data processing

The data collected for the different illumination states were processed using the program dials.stills_process with a target unit cell of *a* = 117.0 Å, *b* = 221.0 Å, *c* = 309.0 Å, *α* = *β* = *γ* = 90° and the space group P2_1_2_1_2_1_. Bragg spots were integrated to the edge of the detector. A Kapton absorption correction due to the conveyor belt of our sample delivery system was applied to each integrated Bragg spot, taking into account the droplet size, tape thickness, tape angle and the position of the diffraction spots on the detector with respect to the crystal position. Before integration, we also performed ensemble refinement of the crystal and detector parameters using the program cctbx.xfel.stripe_experiment, which has been shown to narrow the unit cell distribution and improve the final isomorphous difference maps^56^. Finally, the intensities were merged using the program cctbx.xfel.merge, which applies a per-image resolution cutoff and filtering of the lattices using a unit cell threshold of 1% from the reference model. To merge the reflections, we use the best practices described in ref. ^57^. The unit cells and number of lattices merged for each dataset are tabulated in Extended Data Tables 1 and 2.

Final merged datasets were acquired for the 2F, 3F(50 µs), 3F(250 µs), 3F(500 µs), 3F(730 µs), 3F(1,200 µs), 3F(2,000 µs), 3F(4,000 µs) and 3F(200 ms) states to resolutions between 2.16 and 2.0 Å, obtained by merging between 6,659 and 39,199 lattices (Extended Data Tables 1 and 2). The final merged datasets before model building were also scaled on a per-resolution bin basis to a reference dataset (in this case, the reference dataset is the PS II dataset published in PDB ID code 7RF1)^12^. This allows us to conduct a more accurate comparison of m*F*_obs _− D*F*_calc_ omit maps and 2m*F*_obs _− D*F*_calc_ maps between different datasets.

### Model building and map calculation

Each dataset was refined using a high-resolution PS II structure (1.89 Å) that was published in a previous work (PDB ID code 7RF1)^12^ as the starting point using the program phenix.refine^58^. The refinement is done in several stages. First, the B factors of the starting model are set to 30, and all waters and the atoms of the OEC are removed. An initial rigid body refinement coupled with refinement of *xyz* coordinates and isotropic B factors was done for 15 cycles to adjust the model into the unit cell. Next, the OEC atoms are added back and refined with custom bonding restraints for several cycles. We also use custom bonding restraints for chlorophyll-*a* (to allow correct placement of the Mg relative to the plane of the porphyrin ring) and unknown lipid-like ligands (steric acid) in the refinement. After initial refinement of the OEC + protein complex, waters were added to the model using the phenix.refine water picking protocol as well as manual placement of waters via coot^59^ and doing multiple cycles of refinement.

At this stage, we split the model in the vicinity of the OEC and the OEC itself (only protein and OEC atoms) into multiple components (Extended Data Table 3). The split was done only in parts of chains A/a, C/c and D/d. The rationale and population of the components in each time point used are described in the section Estimating population distribution in each time point. In each dataset, the primary conformer (defined as the intermediate that is advancing from S_3_ to S_0_) is refined using a strategy of reciprocal *xyz* + isotropic B-factor refinement. For the secondary/tertiary components (whose structures are known as they are either in the S_3_ or S_0_ state), only the group B factors are adjusted (group_adp strategy in phenix.refine) to adjust them to the resolution of the dataset. For the remaining part of the model that is not split, regular reciprocal *xyz* refinement and isotropic B-factor refinement are performed in tandem for multiple cycles. All waters (except for the terminal waters ligated to the OEC; that is, W1–W4) were refined as a single component.

The refinement of the OEC in the primary component of the multicomponent model was done using custom restraints that were used to model the S_3_ state. However, for all the time points, we used slightly looser estimated s.d. values for the restraints (0.1 Å for bonds, 10° for angles) to allow the OEC atoms during refinement to move toward where the electron density is optimally modeled and reduce strain in the refinement while at the same time maintaining the overall shape of the cluster. The OEC in the S_0_ state was modeled with restraints used for our previously published S_0_ state structure. The restraints used to model the OEC atoms in the time points have been provided as text files (schemes 1–3 in Supplementary Data).

### Estimating population distribution in each time point

The S-state population distribution in the S_3_→S_0_ transition is a heterogeneous distribution consisting of (1) centers that are advancing from the S_3_ to S_0_ state, (2) centers that are lagging behind by one transition and hence, are advancing from the S_2_ to S_3_ state and (3) centers that have transitioned over to the S_0_ state. While the majority of centers are in category (1), due to the intrinsic inefficiencies (‘misses’) of the Kok cycle in PS II^20^, a certain fraction of centers is in category (2). In addition, after a certain time in the S_3_–S_0_ transition, a substantial number of centers will have formed the stable S_0_ state (category (3)).

Given this context, it is important to account for this population heterogeneity in our structural modeling to obtain accurate electron density maps and models. We do this by splitting up our structural model near the active site region (including the OEC) into multiple components. The primary component in each dataset is category (1), which is the intermediate transitioning from S_3_ to S_0_. The nature of the secondary and tertiary components depends on the dataset under consideration. In each dataset, the coordinates/isotropic B factors of only the primary component are refined (category (1)). The secondary and tertiary component structures are modeled from known or previously deposited structures and only adjusted for resolution using a group B-factor refinement. The identity of the secondary (and tertiary if used) component depends on which time point is being processed. For example, in the 3F(50 µs) dataset, a two-component model is constructed with the 2F(50 µs) model coordinates/B factors being used for the secondary component. In the 3F(1,200 µs) data, we use a three-component model with the secondary and tertiary components being the S_0_ and S_3_ states. The populations for each of the components in the various time points are given in Extended Data Table 3. We used numbers available in the literature to perform a kinetic analysis yielding an estimate of the population distribution. Since populations below 10% are in the noise level for structural refinement, we adjusted our populations to avoid any conformer with such low populations.

The population distribution in each of the metastable S states has been previously determined using the MIMS technique. In our work, the starting 2F state, which is generated by illuminating with two visible lasers with a flash interval of 200 ms, consists of approximately 65% S_3_ state and 35% S_2_ state based on studies conducted on crystals. With the third visible flash, the S_3_→S_0_ transition is initiated. The reader is referred to the extended data in ref. ^4^ for more details on how the S-state populations for each of the flash states were estimated, accounting for miss parameters calculated from XES and MIMS data, and crossillumination (this was negligible at the speed of the tape and deposition frequency of the acoustic droplet ejection that was used in the present study). All the results described in this paper are from monomer I (chains annotated as uppercase in the published structures). Similar trends are observed for monomer II (chains annotated as lowercase).

### Estimating the effect of population on the Mn1–Mn4 distance

The Mn1–Mn4 distance stays elongated until 3F(1,200 µs) in the intermediate undergoing the S_3_**→**S_0_ transition, after which a decrease is seen in the next 3 ms. We tested the robustness of the elongated distance at 3F(1,200 µs) by constructing an alternative hypothesis to explain this observation, postulating that it could be due to two separate populations in the primary component: (1) increased Mn1–Mn4 distance due to misses that form additional S_3_ or (2) decreased Mn1–Mn4 distance (with/without disappearance of O_x_) that is a property of the intermediate undergoing the S_3_**→**S_0_ transition. We modeled this scenario by increasing the S_3_ population from 35 to 55% and decreasing the primary component (with/without O_x_) from 40 to 20% in the 3F(1,200 µs) time point. The resulting refinement gave Mn1–Mn4 distances of 5.14 Å (with O_x_) and 5.09 Å (without O_x_). Both numbers are similar to the distance given in Fig. 3 and within the measurement error. The tests thus show no contraction compared with the S_3_ state and allow us to reject the hypothesis. We reiterate that the S_3_ population estimate of 35% in the 3F(1,200 µs) time point is well established using multiple independent experiments as detailed in the previous section and past publications.

### Estimated positional precision

To estimate the positional precision of the OEC atoms and the surrounding amino acids for each time point, we used the END/RAPID procedure^60^, similar to what was previously employed^6^. Briefly, in this method, we perturb the structure factors by a random amount in between ±(m*F*_obs _− D*F*_calc_). The atomic coordinates of the final model for that time point are also perturbed by a small amount to allow the model to explore greater phase space (only the primary conformer is perturbed). Subsequently, 100 such synthetic datasets are generated for each time point, and they are then each refined separately. From the ensemble of these refined datasets, we can estimate the error associated with the distance metric of interest. The obtained errors should be considered an upper bound as the introduced perturbations in the structure factors are an overestimate of the true errors in the experiment.

Implementation details can be found at https://bl831.als.lbl.gov/END/RAPID/end.rapid/Documentation/end.rapid.Manual.htm.

### m*F*_obs _− D*F*_calc_ difference omit density

All m*F*_obs _− D*F*_calc_ omit maps shown in the manuscript were generated using the phenix.polder program and using the normal omit map coefficients from the output.mtz file (not polder map coefficients)^61^. For peak height calculation wherever stated, we used custom python scripts that average the m*F*_obs _− D*F*_calc_ omit map value about a 0.5-Å radius of the atom of interest.

### Reporting summary

Further information on research design is available in the Nature Portfolio Reporting Summary linked to this article.

## Online content

Any methods, additional references, Nature Portfolio reporting summaries, source data, extended data, supplementary information, acknowledgements, peer review information; details of author contributions and competing interests; and statements of data and code availability are available at 10.1038/s41586-023-06038-z.

## Supplementary information

Supplementary Tables Supplementary Tables 1 and 2.

Reporting Summary

Supplementary Data This zipped folder contains Supplementary Schemes 1–3: Restraints used for modeling the OEC in the S_3_→S_0_ transition.

Supplementary Video 1 Video showing the various changes at the OEC in the S_3_**→**S_0_ transition. High-resolution version available at: https://drive.google.com/file/d/1d4fMKBcYUdenqajlXo3J-a8lU5eaUxe3/view?usp=share_link.

## Figures and Tables

**Extended Data Table 1:**
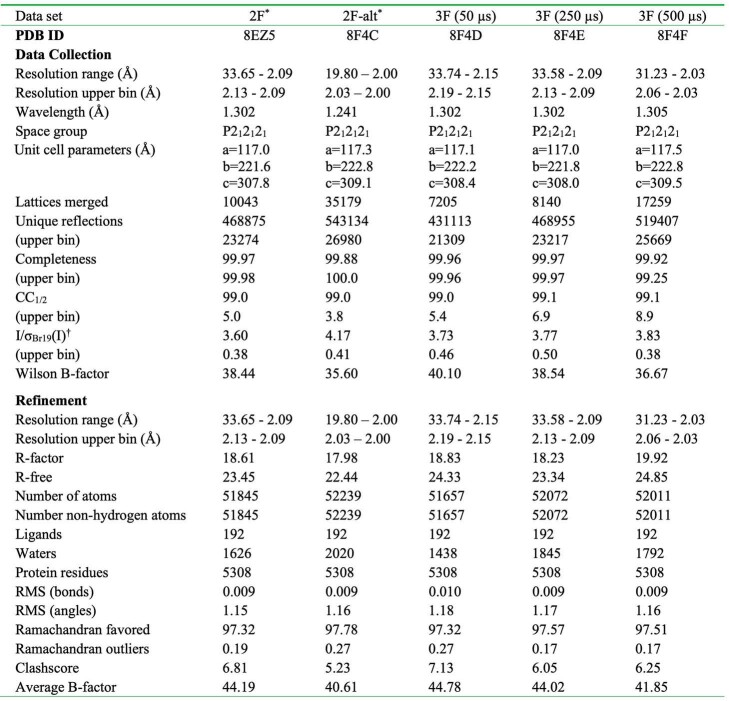
Data collection, merging and refinement statistics (2F, 2F-alternate, 3F(50 µs), 3F(250 µs), 3F(500 µs)).

Note that all reference to the 2F dataset or the S_3_ model in the paper uses the 2.09 Å model. The 2F-alternate model is only provided here for the isomorphous difference maps shown in Fig. 4b. The raw data for the 2F state (PDB code 8EZ5) is the same as that used for the previously reported structural models 6W1V (ref. ^6^) and 7RF8 (ref. ^12^). The data were reprocessed and the atomic structure was re-refined in order to apply the same updated computational methods as employed for the other nine new structures reported here.

**Extended Data Table 2:**
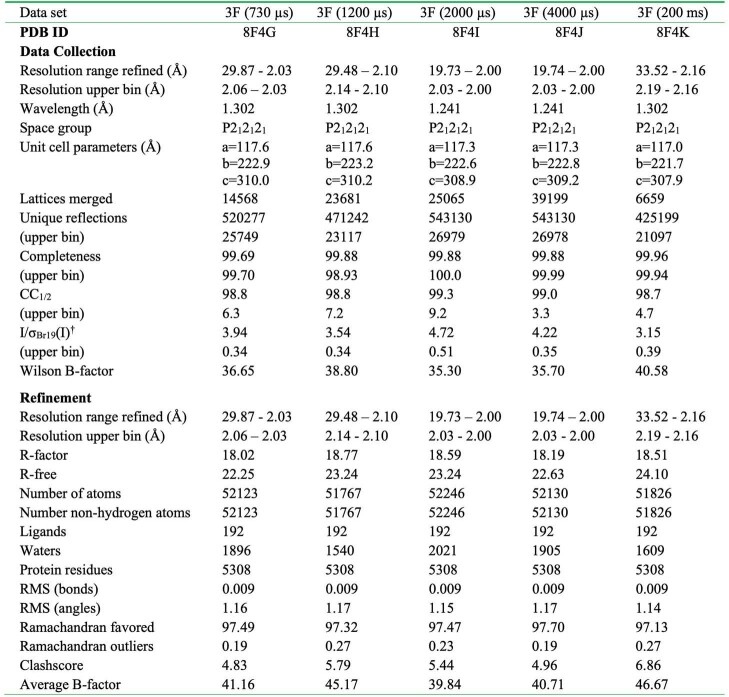
Data collection, merging and refinement statistics (3F(730 µs), 3F(1200 µs), 3F(2000 µs), 3F(4000 µs), 3F(200 ms)).

**Extended Data Table 3:**
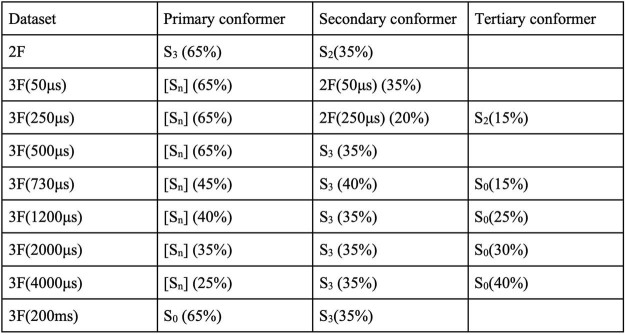
The estimated population distribution and identity of each of the conformers used in the multi-component modeling of the datasets

Refer to the discussion *Estimating population distribution in each timepoint* in Methods for more details regarding the conformers.

**Extended Data Table 4:**
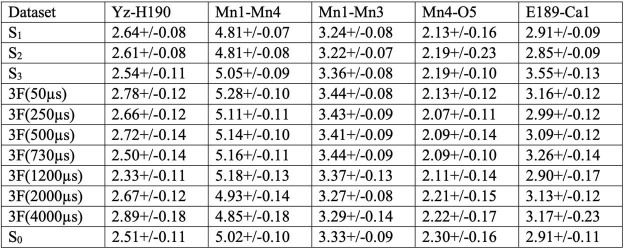
Source data for the plots shown in Figure 3 of the main text.

## Data Availability

The atomic coordinates and structure factors have been deposited in the Protein Data Bank, www.pdb.org (PDB codes 8EZ5 for the 2F data; 8F4D for the 3F(50 μs) data; 8F4E for the 3F(250 μs) data; 8F4F for the 3F(500 μs) data; 8F4G for the 3F(730 μs) data; 8F4H for the 3F(1,200 μs) data; 8F4I for the 3F(2,000 μs) data; 8F4J for the 3F(4,000 μs) data; 8F4K for the 3F(200 ms) data and 8F4C for the 2F-alternate data). The raw X-ray free electron laser data have been deposited in the Coherent X-Ray Imaging Database, www.cxidb.org (ID 215).

## Code availability

The open source programs dials.stills_process, cctbx.xfel GUI, cctbx.xfel.stripe_experiment, cctbx.xfel.merge and legacy program cxi.merge are distributed with DIALS packages available at http://dials.github.io. Instructions and details for using these programs can be found in refs. ^56,57^ with further documentation available at http://cci.lbl.gov/xfel. Figures shown in the paper were rendered using the PyMOL software v.2.5 (ref. ^62^). Custom code used for calculating peak height values is publicly available at https://github.com/asmit3/eden(ref. ^63^).
