# Author Correction: Structural evidence for intermediates during O_2_ formation in photosystem II

**DOI:** 10.1038/s41586-024-07099-4

**Published:** 2024-01-30

**Authors:** Asmit Bhowmick, Rana Hussein, Isabel Bogacz, Philipp S. Simon, Mohamed Ibrahim, Ruchira Chatterjee, Margaret D. Doyle, Mun Hon Cheah, Thomas Fransson, Petko Chernev, In-Sik Kim, Hiroki Makita, Medhanjali Dasgupta, Corey J. Kaminsky, Miao Zhang, Julia Gätcke, Stephanie Haupt, Isabela I. Nangca, Stephen M. Keable, A. Orkun Aydin, Kensuke Tono, Shigeki Owada, Leland B. Gee, Franklin D. Fuller, Alexander Batyuk, Roberto Alonso-Mori, James M. Holton, Daniel W. Paley, Nigel W. Moriarty, Fikret Mamedov, Paul D. Adams, Aaron S. Brewster, Holger Dobbek, Nicholas K. Sauter, Uwe Bergmann, Athina Zouni, Johannes Messinger, Jan Kern, Junko Yano, Vittal K. Yachandra

**Affiliations:** 1 Molecular Biophysics and Integrated Bioimaging Division, Lawrence Berkeley National Laboratory, Berkeley, CA USA; 2 Department of Biology, Humboldt Universität zu Berlin, Berlin, Germany; 3 Molecular Biomimetics, Department of Chemistry — Ångström, Uppsala University, Uppsala, Sweden; 4 Department of Theoretical Chemistry and Biology, KTH Royal Institute of Technology, Stockholm, Sweden; 5 Japan Synchrotron Radiation Research Institute, Hyogo, Japan; 6 RIKEN SPring-8 Center, Hyogo, Japan; 7 Linac Coherent Light Source, SLAC National Accelerator Laboratory, Menlo Park, CA USA; 8 Department of Biochemistry and Biophysics, University of California, San Francisco, CA USA; 9 SSRL, SLAC National Accelerator Laboratory, Menlo Park, CA USA; 10 Department of Bioengineering, University of California, Berkeley, CA USA; 11 Department of Physics, University of Wisconsin–Madison, Madison, WI USA; 12 Department of Chemistry, Umeå University, Umeå, Sweden; 13 Present Address: Institute of Molecular Medicine, University of Lübeck, Lübeck, Germany

**Keywords:** Bioenergetics, Nanocrystallography

Correction to: *Nature* 10.1038/s41586-023-06038-z Published online 3 May 2023

In the version of the article originally published, it was not clarified that the raw data used to obtain one of the refined structural models for the 2F state (PDB 8EZ5) were the same as those used to obtain the previously reported structural models PDB 6W1V and PDB 7RF8. This clarification has now been added as a footnote to Extended Data Table 1.

