# Supplementary material for: Structural evidence for intermediates during O2 formation in photosystem II
Source: Nature. 2023 May 3;617(7961):629–36. doi: 10.1038/s41586-023-06038-z (PMC10191843; doi:10.1038/s41586-023-06038-z)
Supplement: Supplementary file 1 — Supplementary Tables 1 and 2. [file 41586_2023_6038_MOESM1_ESM.pdf]

nature portfolio

https://doi.org/10.1038/s41586-023-06038-z

Supplementary information

Structural evidence for intermediates during O_2 formation in photosystem II

In the format provided by the authors and unedited

Supplementary Information

Structural evidence for intermediates during O_2 formation in Photosystem II

Asmit Bhowmick[1#], Rana Hussein[2#], Isabel Bogacz[1#], Philipp S. Simon[1#], Mohamed Ibrahim[2¶], Ruchira Chatterjee[1], Margaret D. Doyle[1], Mun Hon Cheah[3], Thomas Fransson[4], Petko Chernev[3], In-Sik Kim[1], Hiroki Makita[1], Medhanjali Dasgupta[1], Corey J. Kaminsky[1], Miao Zhang[1], Julia Gätcke[2], Stephanie Haupt[2], Isabela I. Nangca[1], Stephen M. Keable[1], Orkun Aydin[3], Kensuke Tono[5,6], Shigeki Owada[5,6], Leland B. Gee[7], Franklin D. Fuller[7], Alexander Batyuk[7], Roberto Alonso-Mori[7], James M. Holton[1,8,9], Daniel W. Paley[1], Nigel W. Moriarty[1], Fikret Mamedov[3], Paul D. Adams[1,10], Aaron S. Brewster[1], Holger Dobbek[2], Nicholas K. Sauter[1], Uwe Bergmann[11], Athina Zouni[2*], Johannes Messinger[3,12*], Jan Kern[1], Junko Yano[1*], Vittal K. Yachandra[1*]

[1]Molecular Biophysics and Integrated Bioimaging Division, Lawrence Berkeley National Laboratory, Berkeley, CA 94720, USA.

[2]Humboldt-Universität zu Berlin, Department of Biology, 10099 Berlin, Germany.

[3]Department of Chemistry - Ångström, Molecular Biomimetics, Uppsala University, SE 75120 Uppsala, Sweden.

[4]Department of Theoretical Chemistry and Biology, KTH Royal Institute of Technology, Stockholm, Sweden.

[5]Japan Synchrotron Radiation Research Institute, 1-1-1 Kouto, Sayo-cho, Sayo-gun, Hyogo, 679-5198, Japan

[6]RIKEN SPring-8 Center, 1-1-1 Kouto, Sayo-cho, Sayo-gun, Hyogo, 679-5148, Japan

[7]Linac Coherent Light Source, SLAC National Accelerator Laboratory, Menlo Park, CA 94025, USA.

[8]Department of Biochemistry and Biophysics, University of California, San Francisco, San Francisco, California, CA 94158, USA

[9]SSRL, SLAC National Accelerator Laboratory, Menlo Park, CA 94025, USA

[10]Department of Bioengineering, University of California, Berkeley, CA 94720, USA.

[11]Department of Physics, University of Wisconsin–Madison, Madison, WI 53706, USA.

[12]Department of Chemistry, Umeå University, SE 90187 Umeå, Sweden.

[#]These authors contributed equally

[¶]Present address: Institute of Molecular Medicine, University of Lübeck, 23562 Lübeck, Germany.

[*]Correspondence should be addressed to athina.zouni@hu-berlin.de,

johannes.messinger@kemi.uu.se, jyano@lbl.gov, vkyachandra@lbl.gov

This file contains Supplementary Table 1 and 2.

Table of Contents

Supplementary Table 1: Water numbering convention used in this work and relation to water numbering in deposited PDB files 3

Supplementary Table 2: Collection details for various datasets 6

Supplementary Table 1. Water numbering convention used in this work and relation to residue number*s* in pdb files (note that numbers are only given for the first monomer in each model, “x” indicates water not present, for W1-W4 only the refined component is given for 3F(50µs) and later time points

Numbering in manuscript	Channel	2F	3F (50µs)	3F (250µs)	3F (500µs)	3F (730µs)	3F (1200µs)	3F (2000µs)	3F (4000µs)	3F (200ms)
W1		A713	A724	A715	A740	A730	A728	A723	A721	A722
W2		A747	A755	A746	A800	A752	A769	A739	A737	A733
W3		A786	A781	A797	A808	A805	A796	A813	A804	A797
W4		A732	A715	A730	A765	A758	A754	A748	A747	A752
W19	O4	A711	A703	A704	A722	A702	A701	A706	A706	A704
W20	O4	x	x	x	x	x	x	x	C607	C602
W21	Cl1A/B	A712	A714	A724	A745	A727	A716	A728	A735	D505
W22	Cl1A/B	A755	A727	A771	A753	A720	A735	A722	A733	A762
W23	Cl1A/B	A782	A788	A806	A791	A786	A772	A774	A792	A781
W24	Cl1A/B	A798	A790	A802	A809	A821	A792	A827	A810	A780
W25	Cl1A/B	A741	A705	A708	A733	A723	A747	A716	A701	A720
W26	O1A/B	A716	A778	A801	A713	A787	A718	A797	A742	A706
W27	O1A/B	A707	A701	A701	A707	A705	A802	A750	A716	A705
W28	O1A/B, Y_Z	A805	A801	A805	A820	A824	A808	A825	A817	A795
W29	O1A/B, Y_Z	A763	A763	A756	A816	A799	A765	A787	A807	A798
W30	O1A/B, Y_Z	A706	A737	A733	A724	A707	A703	A727	A728	A728
W31	O1A/B	A725	A733	A714	A739	A709	A705	A704	A702	A712
W32	O1A/B	A806	A800	A818	A819	A826	A810	A828	A825	A808
W33	O1A/B	A792	A792	A798	A794	A759	A763	A786	A799	A801
W34	O1B	A749	A770	A719	A706	A716	A719	A715	A754	A715
W35	O1B	C605	C618	C608	C611	C651	C606	C626	C643	C626
W36	O1B	D572	A721	D528	D567	D546	D560	D526	A739	A750
W37	O1B	U201	U201	U201	U209	U202	U210	U203	U203	U202
W38	O1B	V348	V340	V345	V346	V348	V345	V354	V351	V337
W39	O1A/B	A799	A798	A770	A810	A807	A807	A766	A789	A806
W40	Cl1A	A705	A702	A713	A732	A713	A702	A707	A709	A703
W41	Cl1A	D526	D513	D530	D510	D525	D503	D563	D520	D529
W42	Cl1A	A791	A793	A799	A812	A819	A797	A798	A808	A792
W43	Cl2	A764	A732	A790	A763	A788	A722	A735	A711	A771
W44	Cl2	A800	A787	A810	A801	C734	A770	C739	C721	A787
W45	Cl2	C622	C674	C618	C645	C648	C679	C628	C640	C641
W46	Cl2	C682	C647	C686	C670	C697	C662	C714	C695	C675
W47	Cl2	C668	C651	C677	C643	C686	C660	C662	C685	C676
W48	O4	A748	A753	A721	A805	A765	A789	A772	A755	A742

Supplementary Table 1 continued

Numbering in manuscript	Channel	2F	3F (50µs)	3F (250µs)	3F (500µs)	3F (730µs)	3F (1200µs)	3F (2000µs)	3F (4000µs)	3F (200ms)
W49	O4	A761	A734	A750	A736	A748	A741	A710	A714	A718
W50	O4	C690	C696	C720	C682	C732	C720	C748	C741	C691
W51	O4	C704	C691	C718	A804	C733	C707	A824	C728	C711
W52	O4	A740	A709	A728	A720	A736	A714	A738	A712	A713
W53	O4	C679	C672	C689	C696	C669	C678	C711	C698	C648
W54	Y_Z	A784	A757	A788	A786	A817	A806	A821	A796	A791
W55	Y_Z	A730	A720	A764	A738	A763	A717	A717	A764	A719
W56	Y_Z	C611	C626	C649	C609	C608	C614	C607	C616	C605
W57	Y_Z	A715	A718	A707	A705	A704	A709	A730	A704	A739
W58	Y_Z	A788	A769	A794	A775	A791	A790	A785	A774	A760
W59	Cl1B	A766	A766	A783	A813	A780	A767	A702	A703	A774
W60	Cl1B	D580	D576	D545	D587	D592	D572	D593	D550	D563
W61	Cl1A/B	A771	A776	A772	A790	A756	A780	A775	A734	A789
W62	Cl1A/B	A718	A716	A738	A747	A745	A704	A731	A724	A714
W66	Cl1B	D522	D549	D521	D548	O339	D513	D515	D533	D541
W67	Cl1B	O312	O333	O335	D581	D561	O358	O352	D569	D554
W68	Cl1B	D563	D563	D595	O376	D603	D590	D598	D588	D589
W69	Cl1B	D515	O309	D522	O328	O315	O313	O346	O311	O308
W70	Cl1B	D601	D592	D616	D618	D626	D599	D621	D611	D610
W71	O4	A756	A743	A749	A788	A728	A731	A768	A723	A724
W72	O4	D594	D581	D602	D605	D612	D580	D606	D591	D597
W73	O4	U223	U220	U238	U226	U236	U232	U239	U231	U231
W74	O4	O324	O307	C625	O326	C616	C615	O315	C627	C625
W75	O4	U216	U209	U225	O340	O316	U214	U221	O349	U217
W76	O1B	C618	C656	V350	C727	C744	x	C738	C749	V336
W77	O1B	V350	V343	V350	V364	V364	V348	V358	V359	X
W101	O1B	D527	U208	U226	D530	D537	D556	D548	D530	D560
W102	O1B	B857	B842	B868	B995	B886	B846	B863	B867	B832
W103	O1A	C650	C639	C690	C603	C679	C651	C681	C647	C663
W104	O1A	C725	C697	C730	C735	C754	C730	C763	C756	C721
W106	O1A	C716	C694	C728	C724	C746	C722	C761	C743	C722
W107	O1A	C693	C649	C712	C663	C658	C642	C690	C664	C702
W117	Cl1A	D597	D589	D608	D610	D619	D596	D618	D605	D601
W119	Cl1A	A727	A723	A737	A726	A710	A734	A737	A708	A717
W121	Cl1A	O370	O353	O380	O400	O368	O360	O363	O367	O368
W122	Cl1A	O335	O339	O378	O396	O336	O362	O377	O366	O350
W125	Cl1A	O354	O337	D579	D571	O352	D542	O331	D564	O327
W126	Cl1A	D588	O360	D582	D599	D615	D579	D600	D566	D594
W127	Cl1A	D582	D570	D605	D579	A793	A784	D583	D561	D572
W129	Cl1A	A783	A754	A784	D598	D610	A746	A800	A805	A759
W145	Cl1A	x	x	x	x	x	x	x	x	x
W150	Cl1A	x	x	x	x	x	x	x	x	x
W151	O1B	x	x	x	x	x	x	x	x	C714
152	Cl1A	A736	A747	A727	A718	A714	A712	A718	A725	A726
153	Cl1A	O322	O334	O334	O355	O334	O325	O338	O352	O316
154	Cl1A	O356	O335	O355	O347	O357	O338	O360	O360	O353
160	Cl1B	B794	B771	B799	B958	B794	B773	B793	B778	B792
161	Cl1B	D513	D503	D520	D512	D516	D514	D505	D505	D511
162	Cl1B	B725	B706	B762	B978	B727	B710	B777	B737	B745

Supplementary Table 1 continued

Numbering in manuscript	Channel	2F	3F (50μs)	3F (250μs)	3F (500μs)	3F (730μs)	3F (1200μs)	3F (2000μs)	3F (4000μs)	3F (200ms)
163	Cl1B	D538	D517	O322	D539	O320	D536	O336	O320	D528
164	Cl1B	O314	O314	O318	O331	O328	O326	O333	O318	O304
165	Cl1B	B756	B750	B775	B945	B787	B751	B798	B804	B737
166	Cl1B	B747	B713	B761	B1014	B743	B709	B776	B770	B733
167	Cl1B	D586	D580	D568	D600	D606	D591	D601	D593	D596
168	Cl1B	B782	B749	O343	O345	B759	B732	O316	O331	B740
169	Cl1B	B840	O362	B701	B931	x	B701	B880	B860	x
170	Cl1B	O352	O338	O375	O378	O360	O336	O366	B830	O335
180	O4	O317	O323	O331	O330	O332	O328	O350	O313	U220
181	O4	U208	U207	U215	U213	U211	U221	U220	U211	U221
182	O4	O373	O363	U245	U233	U240	U233	U244	U239	U232
183	O4	U227	U218	U241	U225	U227	U230	U237	U230	U225
184	O4	U215	U216	U233	U205	U221	U213	U230	U219	U214
190	O1B	D516	D571	D571	D573	D563	D594	D514	D542	D564
191	O1B	D603	D591	D610	D615	D625	D600	D617	D609	D609
192	O1A	C675	C648	C651	C658	C683	C723	C645	C659	C708
193	O1A	C637	C634	C628	C679	C615	C650	C656	C666	C611
194	O1A	C689	C653	C620	C684	C638	C685	C720	C676	C655
195	O1A	x	V324	V330	V328	V305	V332	V305	V323	V342
196	O1A	C711	C658	C719	C729	C703	C713	x	C748	C718
197	O1A	C676	C654	C707	C638	C659	C712	C659	C700	C684

Supplementary Table 2: Collection details for the various datasets.

**Dataset**	**Facility**	**Proposal**	**Detector Configuration**	**Rep rate [Hz]**	**Beam energy [keV]**	**X-ray spot [µm]**	**Pulse length [fs]**	**Beam power [mJ]**
2F	MFX, LCLS	MFXLU3417	Rayonix MX340 (3-by-3 binning)	20	9.5	3	35	2-3
2F-alternate	BL2, SACLA	2018B8089	Rayonix MX300 (3-by-3 binning)	20	10.0	1.5	10	0.48
3F(50µs)	MFX, LCLS	MFXLU3417	Rayonix MX340 (3-by-3 binning)	20	9.5	3	35	3-3.5
3F(250us)	MFX, LCLS	MFXLU3417	Rayonix MX340 (3-by-3 binning)	20	9.5	3	35	2-4
3F(500µs)	MFX, LCLS	MFXLX4319	Rayonix MX340 (3-by-3 binning)	20	9.5	3	35	2-4
3F(730µs)	MFX, LCLS	MFXLS1016	Rayonix MX170 (2-by-2 binning)	10	9.5	3	35	2-4
3F(1200µs)	MFX, LCLS	MFXLV7018	Rayonix MX340 (3-by-3 binning)	20	9.5	3	35	3-4
3F(2000µs)	BL2, SACLA	2018B8089	Rayonix MX300 (3-by-3 binning)	20	10.0	1.5	10	0.48
3F(4000µs)	BL2, SACLA	2018B8089	Rayonix MX300 (3-by-3 binning)	20	10.0	1.5	10	0.48
3F(200ms)	MFX, LCLS	MFXLU3417	Rayonix MX340 (3-by-3 binning)	20	9.5	3	35	2-4
