# Supplementary material for: Structural evidence for intermediates during O2 formation in photosystem II
Source: Nature. 2023 May 3;617(7961):629–36. doi: 10.1038/s41586-023-06038-z (PMC10191843; doi:10.1038/s41586-023-06038-z)
Supplement: Supplementary file 2 — Reporting Summary [file 41586_2023_6038_MOESM2_ESM.pdf]

nature portfolio

Corresponding author(s): Junko Yano

Last updated by author(s): Mar 14, 2023

Reporting Summary

Nature Portfolio wishes to improve the reproducibility of the work that we publish. This form provides structure for consistency and transparency in reporting. For further information on Nature Portfolio policies, see our Editorial Policies and the Editorial Policy Checklist.

Statistics

For all statistical analyses, confirm that the following items are present in the figure legend, table legend, main text, or Methods section.

n/a	Confirmed	
☐	☒	The exact sample size (*n*) for each experimental group/condition, given as a discrete number and unit of measurement
☐	☒	A statement on whether measurements were taken from distinct samples or whether the same sample was measured repeatedly
☒	☐	The statistical test(s) used AND whether they are one- or two-sided<br>*Only common tests should be described solely by name; describe more complex techniques in the Methods section.*
☒	☐	A description of all covariates tested
☒	☐	A description of any assumptions or corrections, such as tests of normality and adjustment for multiple comparisons
☐	☒	A full description of the statistical parameters including central tendency (e.g. means) or other basic estimates (e.g. regression coefficient) AND variation (e.g. standard deviation) or associated estimates of uncertainty (e.g. confidence intervals)
☒	☐	For null hypothesis testing, the test statistic (e.g. *F*, *t*, *r*) with confidence intervals, effect sizes, degrees of freedom and *P* value noted<br>*Give P values as exact values whenever suitable.*
☒	☐	For Bayesian analysis, information on the choice of priors and Markov chain Monte Carlo settings
☒	☐	For hierarchical and complex designs, identification of the appropriate level for tests and full reporting of outcomes
☒	☐	Estimates of effect sizes (e.g. Cohen's *d*, Pearson's *r*), indicating how they were calculated

Our web collection on statistics for biologists contains articles on many of the points above.

Software and code

Policy information about availability of computer code

Data collection	We used the live version of psana (ana-current) provided by SLAC and development versions of open source programs dials.stills_process, the cctbx.xfel GUI and cxi.merge, which are distributed with DIALS packages (3.13.0) publicly available at http://dials.github.io, for data collection.
Data analysis	We used development versions of open source programs dials.stills_process, the cctbx.xfel GUI and cxi.merge, which are distributed with DIALS packages (3.13.0) publicly available at http://dials.github.io and END/RAPID structure factor modification software available at http://bl831.als.lbl.gov/END/RAPID/. We also used Phenix structure refinement software available at https://phenix-online.org (version 1.19.2-4158), Coot structure visualization and modification software version 0.8.8-pre, and PyMol structure visualization and figure rendering software version 2.5. Any custom code used for this publication are publicly available at https://doi.org/10.5281/zenodo.7734707.

For manuscripts utilizing custom algorithms or software that are central to the research but not yet described in published literature, software must be made available to editors and reviewers. We strongly encourage code deposition in a community repository (e.g. GitHub). See the Nature Portfolio guidelines for submitting code & software for further information.

Data

Policy information about availability of data

All manuscripts must include a data availability statement. This statement should provide the following information, where applicable:

- Accession codes, unique identifiers, or web links for publicly available datasets
- A description of any restrictions on data availability
- For clinical datasets or third party data, please ensure that the statement adheres to our policy

The atomic coordinates and structure factors have been deposited in the Protein Data Bank, www.pdb.org (PDB code 8EZ5 for the 2F data; 8F4D for the 3F(50µs) data; 8F4E for the 3F(250µs) data; 8F4F for the 3F(500µs) data; 8F4G for the 3F(730µs) data; 8F4H for the 3F(1200µs) data; 8F4I for the 3F(2000µs) data; 8F4J for the 3F(4000µs) data; 8F4K for the 3F(200ms) data and 8F4C for the 2F-alternate data). The raw XFEL data has been deposited in the Coherent X-ray Imaging Database (CXIDB, www.cxidb.org), accession number 215. Source data are provided with the paper.

Human research participants

Policy information about studies involving human research participants and Sex and Gender in Research.

Reporting on sex and gender	No human research involved
Population characteristics	No human research involved
Recruitment	No human research involved
Ethics oversight	No human research involved

Note that full information on the approval of the study protocol must also be provided in the manuscript.

Field-specific reporting

Please select the one below that is the best fit for your research. If you are not sure, read the appropriate sections before making your selection.

☒ Life sciences ☐ Behavioural & social sciences ☐ Ecological, evolutionary & environmental sciences

For a reference copy of the document with all sections, see nature.com/documents/nr-reporting-summary-flat.pdf

Life sciences study design

All studies must disclose on these points even when the disclosure is negative.

Sample size	Sample size is stated for all data sets in the Method section and Supplementary Data Table 1. Sample size was chosen to ensure at least 10 independent observations for each structure factor upto the resolution limit reported.
Data exclusions	During merging of structure factors, lattices that deviated by 1% or more from the reference unit cell were rejected.
Replication	Diffraction data was collected once for each state. We simulate the experimental errors possible using the END/RAPID approach as described in the Methods section. The END/RAPID approach gives us n=100 different synthetic datasets for which we performed independent refinements, confirming the results and giving an error estimate for atom positions of interest.
Randomization	Different illumination states were interleaved as much as possible during data collection within the constraints of the XFEL experiment. Data and refinement quality was assessed based on CC1/2 and R-free statistics and the datasets were randomly subsampled to generate these statistics. Other randomizations were not relevant for the XFEL experiment.
Blinding	Investigators were not blinded to group allocation during data collection. Blinding was not relevant to this study as data for all different states studied was collected interleaved, ensuring similar experimental conditions. Subsequent data treatment was also performed in the same way for all data sets.

Reporting for specific materials, systems and methods

We require information from authors about some types of materials, experimental systems and methods used in many studies. Here, indicate whether each material, system or method listed is relevant to your study. If you are not sure if a list item applies to your research, read the appropriate section before selecting a response.

Materials & experimental systems

n/a	Involved in the study
☒	☐ Antibodies
☒	☐ Eukaryotic cell lines
☒	☐ Palaeontology and archaeology
☒	☐ Animals and other organisms
☒	☐ Clinical data
☒	☐ Dual use research of concern

Methods

n/a	Involved in the study
☒	☐ ChIP-seq
☒	☐ Flow cytometry
☒	☐ MRI-based neuroimaging
